# Novel RNA-targeted therapies for hereditary ATTR amyloidosis and their impact on the autonomic nervous system

**DOI:** 10.1007/s10286-019-00626-8

**Published:** 2019-08-09

**Authors:** Isabel Conceição

**Affiliations:** 1 Department of Neurosciences and Mental Health, CHULN, Hospital de Santa Maria, Av. Prof. Egas Moniz, 1649-035 Lisbon, Portugal; 2 Faculdade de Medicina-IMM, Universidade de Lisboa, Lisboa, Portugal

**Keywords:** Antisense, Interfering RNA, Oligonucleotides, ATTR amyloidosis, Autonomic dysfunction

## Abstract

**Purpose:**

Transthyretin-mediated hereditary amyloidosis (hATTR amyloidosis) is a multisystemic disease with heterogeneous clinical presentation. Hallmarks of the disease are sensory-motor and autonomic neuropathy and cardiomyopathy. Two disease-modifying drugs, inotersen (an antisense oligonucleotide) and patisiran (a small interfering RNA agent), were recently approved for the treatment of hATTR polyneuropathy. We here review the results of the RNA-targeted therapy clinical trials with special emphasis on the endpoints measuring autonomic symptoms and function.

**Methods:**

Literature review. We used the terms “autonomic neuropathy”, “dysautonomia”, “autonomic symptoms”, “oligonucleotides”, “inotersen” and “patisiran” in patients with hATTR amyloidosis.

**Results:**

In the NEURO-TTR (inotersen) clinical trial, the modified NIS+7 score (mNIS+7) remained stable in 36% of the patients in the inotersen arm (defined as a change of less than 2 points), and 50% of patients had improved quality of life (Norfolk-QOL-DN score) after 15 months. In the APOLLO patisiran trial, 74% of the patients showed stabilization of the neuropathy, defined as a < 10 points increase on mNIS+7, and 51% of patients showed an improvement of quality of life (Norfolk QOL-DN), favoring patisiran at 18 months. Patients on patisiran had a reduced burden of autonomic dysfunction as measured by the COMPASS-31, and a stabilization of nutritional status, suggesting an effect on gastrointestinal autonomic function.

**Conclusions:**

Clinical trials of inotersen and patisiran showed that these agents were able to halt the progression of the disease, with some patients even reducing the burden of polyneuropathy, and improving qualify of life. The information on their impact on autonomic parameters is limited, warranting further dedicated studies.

## Introduction

Transthyretin-mediated hereditary amyloidosis (hATTR amyloidosis) is a multisystemic debilitating, progressive and life-threatening disease, caused by the deposition of misfolded transthyretin protein as amyloid fibrils at the extracellular space of the somatic and autonomic peripheral nervous system, cardiac, renal, ocular and central nervous systems [1, 2]. Worldwide prevalence estimates indicate that approximately 50,000 people have been diagnosed with hATTR amyloidosis [3].

hATTR amyloidosis is a highly heterogeneous disease with a broad genotype–phenotype presentation that may present with a wide range of clinical manifestations [2, 4]. The best known phenotypes are the neuropathic and cardiac ones, with the V30M (p.V50M) mutation being the most common TTR variant described worldwide, comprising 73% of all hATTR amyloidosis cases [5]. hATTR V30M amyloidosis is mainly associated with a sensory-motor and autonomic neuropathy, although 5% of patients also present with a pure cardiomyopathy [5].

Sensory motor and autonomic neuropathy is the hallmark of the early-onset hATTRV30M amyloidosis, although it is also reported in non-V30M hATTR amyloidosis [6]. Autonomic symptoms included pupillary, secretomotor, peripheral and cardiac vasomotor, gastrointestinal, urinary, and sexual dysfunction with an important impact on disability and quality of life [7]. Dysautonomia can occur before the somatic peripheral nerve manifestations, being one of the first signs of early-onset V30M hATTR amyloidosis [2].

Gastrointestinal (GI) manifestations are prominent in most cases, being one of the clinical manifestations mostly related to the poor quality of life of these patients. [8]. GI involvement is reported in V30M (69%) as well as in non-V30M (56%) hATTR amyloidosis patients after 5 years of disease duration; however, for some mutations with mainly cardiac phenotype, the frequency of GI symptoms ranged from only 10 to 27% [9]. Unintentional weight loss, nausea, vomiting and constipation alternating with diarrhea are common during early-stage disease, while malabsorption, severe chronic diarrhea, severe malnutrition and cachexia which usually occurs during late-stage disease [8]. The pathogenesis of GI involvement is not fully understood, but autonomic dysfunction, which seems to be related to motility disturbances [10], depletion of the enteric nerves and endocrine cells, has also been described [11].

Cardiovascular autonomic involvement is an important autonomic manifestation in hATTR amyloidosis attributed to sympathetic and parasympathetic dysfunction that may induce reduced heart rate variability (HRV) [12], impairment of blood pressure regulation and orthostatic hypotension, cardiac conduction abnormalities and complex ventricular arrhythmias, associated with an increased risk of sudden death [12].

Parasympathetic dysfunction also contributes to genitourinary dysfunction, starting from urinary retention with incomplete bladder emptying and urinary urgency progressing to urinary incontinence [4, 12]. Urinary retention and recurrent urinary tract infections contribute to a significant associated morbidity with an increased risk of urosepsis that may be the cause of death in some patients [4]. In men, erectile dysfunction proceeding to impotence is one of the first findings in the course of the disease, contributing to a significant decrease in quality of life [4].

Evaluating the autonomic function is an important issue in hATTR amyloidosis, acknowledging an early diagnosis, monitoring modifying disease therapeutics, and predicting the prognosis [12, 13].

Recent studies have shown that TTR-specific oligonucleotides, either small interfering RNAs (patisiran) or antisense oligonucleotides (inotersen), can reduce TTR levels of the mutated and wild-type, with significant clinical efficacy in the progression of hATTR amyloid neuropathy over time [14, 15].

In this review, we address the therapeutic value of the recently approved RNA-targeted therapies (inotersen and patisiran) on hATTR amyloidosis, with special emphasis on autonomic dysfunction.

### Clinical vignette

A 47-year-old Portuguese male, with a past history of tuberculosis and moderate alcoholic and smoking habits, was diagnosed as hATTRV30 amyloidosis 4 years after the first symptoms, characterized by diarrhea and unintentional weight loss of 20 kg, followed 2 years later by burning feet and sexual dysfunction. At the time of diagnosis, a length-dependent sensory-motor and autonomic neuropathy was confirmed and molecular testing disclosed a V30M TTR mutation (p.Val50Met).

At the time of diagnosis, at age 42, the patient was able to walk unaided, but with bilateral foot drop [stage 1 of disease, Polyneuropathy Disability Score (PNDs) 2], and had severe diarrhea (more than 8 times/day) with nocturnal fecal incontinence, urinary retention with effort incontinence, sexual dysfunction and renal involvement with a non-nephrotic proteinuria. An autonomic cardiac involvement was described with a 2nd degree AV block, decreased heart rate variability, and an inverted dipper pattern on ambulatory blood pressure monitoring. No amyloid load was seen at DPD scintigraphy score and there was a normal echocardiogram.

The disease progressed to stage 2 with PNDs 3b, despite a TTR stabilizer treatment during 1.5 years. The stabilizer was withdrawn and the patient was enrolled on a double-blind clinical trial with an oligonucleotide. At the time of inclusion, besides the sensory motor neuropathy, a severe autonomic involvement was present, with sexual impotence, severe diarrhea (more than 10 times/day, predominantly nocturnal) with fecal incontinence, a neurogenic bladder with recurrent urinary tract infections, urinary incontinence and symptomatic postural hypotension with syncope.

GI manifestations improved gradually until the present, with a significant decrease in the frequency of diarrhea (3–4 times/day), particularly during the night period, which resulted in a meaningful improvement in sleep quality. Symptoms of neurogenic bladder and sexual dysfunction remain stable. The patient experienced the disappearance of syncope, maintaining some orthostatic dizziness but with no need for additional symptomatic therapy. The patient remains in stage 2 with a PNDs IIIb after 42 months of therapy.

This case illustrates the typical clinical picture of an early-onset hATTR V30M amyloidosis patient whose symptomatology begins with autonomic dysfunction with marked gastrointestinal involvement, followed by somatic fiber dysfunction (sensory-motor neuropathy). In this case, the diagnostic delay had a significant impact on his functional status, conditioning a non-response to the first disease-modifying therapy instituted, due to the advanced stage of the disease. Oligonucleotide therapy allowed a significant reduction of gastrointestinal symptoms and stabilization of sensory-motor neuropathy, with a significant improvement in the quality of life throughout the treatment.

## End-point measures and safety assessments of autonomic function on clinical trials with RNA-targeted therapies

Several tools have been used to evaluate the autonomic function in hATTR amyloidosis [12, 16], but to date there is no consensus as to what is the ideal tool or toolkit for assessing autonomic dysfunction. Recent clinical trials with oligonucleotides used different tools to evaluate the progression of neuropathy, including some autonomic measurements. The different endpoint measurements used will be described below, focusing on autonomic tools.

### Modified Neuropathy Impairment Score Plus 7 (mNIS+7) assessment

The modified Neuropathy Impairment Score Plus 7 (mNIS+7) is a score composed of signs of polyneuropathy and neurophysiological tests that has been validated as a metric of hATTR amyloidosis disease burden and neuropathy progression [17]. The score aims to represent broadly and adequately the diversity and severity of the polyneuropathy impairments, evaluating both the function of small and large somatic fibers as well as the non-myelinated autonomic fibers [17].

Concerning small fiber sensation, a quantitative sensory test (QST) assessment to estimate the touch-pressure (TP) and heat as pain (HP5) sensation loss over the surface of the body is used, with scoring based on percentile reference value at each time [17]. The measurement of the autonomic function depends on the clinical trial in question: heart rate response to deep breathing (HRDB) was used in the Neuro-TTR (inotersen) study [17], and postural blood pressure drop (orthostatic hypotension-OH) in the APOLLO (patisiran) study [15, 18], with the rationale that HRDB has limitations for patients with a pacemaker or cardiac arrhythmias.

In the Neuro-TTR trial (inotersen), progression of sensory-motor and autonomic neuropathy was assessed by the mNIS+7 total score with the maximum abnormality of 346 points. The mNIS+7 total score comprises eight components, four belonging to the total NIS (max. abnormality = 141.4 points) while the other four are heart rate deep breathing (3.7 points), nerve conduction tests (18.6 points), touch–pressure sensation (40 points), and heat–pain sensation (40 points). Scoring of HRDB is expressed as normal deviates (0–3.72) based on healthy-subject parameters, an increase in score indicating a decline in function [14, 17].

Neuropathy progression in the APOLLO trial was assessed by mNIS+7 score (max 304 points) with five subcomponents: mNIS+7 motor (192 points), QST (80 points), reflexes (20 points), NCS (10 points), and postural blood pressure BP (2 points). Scoring of the postural blood pressure is based on grading of function: normal (< 95th percentile) = 0 points; mildly reduced (≥ 95th– < 99th percentile) = 1 point; and very reduced (≥ 99th percentile) = 2 points [15, 18].

### Norfolk Quality of Life Questionnaire diabetic neuropathy (QOL-DN)

The Norfolk Quality of Life Questionnaire diabetic neuropathy (Norfolk QOL-DN) is a 35-item patient self-reporting questionnaire of quality of life able to capture symptoms of both peripheral and autonomic neuropathy related to hATTR disease severity [19]. A recent study showed that, in the early stages of the disease, autonomic dysfunction overlaps the interference in daily life activities due to motor or sensory impairment [19].

Five domains are grouped according to small fiber, large fiber, and autonomic nerve fiber function and symptoms and activities of daily living. Scoring was based on one composite score (total QOL, maximum 136 points) and five subdomain scores: physical functioning/large fiber neuropathy (56 points), activities of daily living (20 points), symptoms (32 points), small fiber neuropathy (16 points), and autonomic neuropathy (12 points). Autonomic function is captured according to the patient’s prompt response and classified from always (5) to never (0) on a 5-point scale. Higher scores reflect a poorer quality of life [20]. Both trials used the Norfolk QOL-DN as a measurement of quality of life.

### Composite Autonomic Symptom Score (COMPASS-31)

The Composite Autonomic Symptom Score (COMPASS-31) is a self-assessment instrument of autonomic symptoms and a function tool to assess and grade symptoms related to autonomic dysfunction [21]. Validated for small fiber neuropathy populations, it was designed to provide a global autonomic severity score and domain scores [21].

The COMPASS-31 comprises six different autonomic domains: three questions for orthostatic intolerance, three for vasomotor symptoms, four for secretomotor function, twelve for the gastrointestinal domain, three for bladder functioning, and five for pupillomotor function. Each question has a weighting factor that results in a maximum impairment score for each domain, with a total maximum impairment of 100 points [21].

The COMPASS-31 was only used in the APOLLO Trial (patisiran) as a secondary exploratory endpoint to assess autonomic involvement [15].

### Nutritional status measurements

The modified BMI (mBMI) [BMI × albumin level (g/dL)] and the body mass index (BMI) (kg/m^2^) are recognized measurements of nutritional status and recognized effective tools for monitoring hATTR disease progression. They are closely related to survival, duration of gastrointestinal disturbances, malabsorption, and functional capacity [22]. mBMI was used as an exploratory secondary endpoint in the APOLLO trial (patisiran) [15], and BMI was evaluated in subgroup analyses in the Neuro-TTR (inotersen) study [14].

## Effectiveness of RNA-targeted therapies on autonomic function

### Inotersen

Inotersen (Tegsedi, Akcea Therapeutics) is an antisense oligonucleotide designed to bind to wild-type and mutant transthyretin RNA transcripts, resulting in their degradation in the nucleus of the hepatocyte by ribonuclease H, with subsequent reduction of the amount of mRNA available for translation. Inotersen is administered by a subcutaneous route once weekly. It has recently received marketing authorization approval from the European Medicines Agency (EMA) for the treatment of stage 1 or stage 2 polyneuropathy in adult patients with hATTR amyloidosis, and regulatory approval from the US FDA for the treatment of the polyneuropathy of hATTR amyloidosis in adults.

These approvals were based on results from the phase III Neuro-TTR study in patients with hATTR amyloidosis with polyneuropathy during 15 months [14]. Primary endpoints of the trial were changes from baseline to week 66 in the mNIS+7 composite score, and in the total score on the Norfolk QOL–DN questionnaire.

Inotersen showed a slowing of the progression of polyneuropathy (assessed by the mNIS+7 score) relative to placebo, and stabilized neuropathy-related quality of life as measured by the Norfolk QOL-DN score.

On average, patients who received inotersen had an increase of 5.8 points (95% CI = 1.6–10.0) from baseline in the mNIS+7 [vs. 25.5 points (95% CI = 20.2–30.8) with placebo] and of 1.0 point (95% CI = − 3.2–5.2) in the Norfolk QOL-DN score [vs. 12.7 points (95% CI = 7.4–17.9) with placebo] significantly favoring inotersen by the end of the intervention period [14].

Concerning the efficacy of treatment in the mNIS+7 component and subcomponent scores, and in the Norfolk QOL-DN domains, the treatment effect favors inotersen in all domains, with a significantly smaller difference versus placebo for the autonomic and small fiber domains [14].

In mNIS+7, the heat–pain domain showed a reduction of 3.54 points (95% CI = − 5.67–1.42) and the HRDB subcomponent only showed a decrease of 0.1 point (95% CI = 0.33–0.12) from baseline. In the Norfolk QOL-DN, the autonomic and small fiber domains are those with a minor evidence of therapeutic efficacy, with a small increase of 0.12 points in the small fiber domain (95% CI = − 0.95–1.19) from baseline, and a non-significant decrease of baseline of − 0.59 points (95% CI = − 1.37–0.18) in the autonomic domain [14]. Thirty-three (36%) of the patients, showed no change from baseline in the inotersen arm and 50% had improved quality of life (Norfolk QOL-DN score) after 15 months.

A subgroup analyses of BMI showed a trend in favor of inotersen to slow weight loss (least-squares mean difference, 0.50 points; *P* = 0.51) [14].

### Patisiran

Patisiran (Onpattro, Alnylam Pharmaceuticals) is a small interference RNA administered by intravenous infusion every 3 weeks, targeting hepatic production of mutant and wild-type TTR, resulting in catalytic RISC-medicated mRNA degradation, with a substantial reduction of the amount available for translation.

Patisiran recently received regulatory approval from the US FDA for the treatment of the polyneuropathy of hATTR amyloidosis in adults, and was approved by the EMA for the treatment of hATTR amyloidosis in adults with stage 1 or stage 2 polyneuropathy.

The APOLLO study was a phase 3 double-blind clinical trial that assessed the safety and efficacy of patisiran on patients with hATTR amyloidosis with polyneuropathy during 18 months [15].

The primary endpoint was the change from baseline to 18 months in the mNIS+7. The quality of life (Norfolk QOL-DN) questionnaire and the motor strength, disability [score on the Rasch-built Overall Disability Scale (R-ODS)], gait speed (10-m walk test), nutritional status (mBMI), and patient-reported autonomic symptoms (Composite Autonomic Symptom Score 31) were considered exploratory secondary endpoints [15].

Seventy percent of patients on the patisiran arm reported stable or improved neuropathy stage [odds ratio (OR) = 39.9, 95% CI = 11.0–144.4 vs. placebo], and 56% of patisiran patients showed neurological improvement by mNIS+7 score (OR = 10.0, 95% CI = 4.4–22.5 vs. placebo). The treatment effect was significant for all subgroups and components of the mNIS+7. The sensory domain measured by QST (small fiber function) showed a reduction of 13 points (95% CI = − 16.3 to − 9.8), and the postural BP subcomponent showed a decrease of 0.3 points (95% CI = − 0.5 to − 0.1) from baseline [15].

The change from baseline in the Norfolk QOL-DN score was significantly lower with patisiran than with placebo at 18 months, indicating better quality of life in the drug arm. At 18 months, 51% of the patients who received patisiran had an improvement (decrease from baseline at 18 months) in the Norfolk QOL-DN score, as compared to 10% of those who received placebo [15]. Consistent effects in favor of patisiran were noted in Norfolk QOL-DN scores across all individual domains, namely physical functioning/larger fiber, symptoms and autonomic domains. The improvement in QOL at 18 months in patients on patisiran was related to strength, digestive health, ability to perform everyday activities and steadiness on their feet [23].

The change from baseline in the COMPASS 31 score was significantly lower with patisiran, with a decrease of − 5.3 ± 1.3 points as compared to 2.2 ± 1.9 with placebo (least-squares mean difference, − 7.5 points; *P* < 0.001) at 18 months, indicating less autonomic involvement.

Patients on patisiran showed a decreased of 3.7 points of mBMI compared with a decreased of − 119.4 points in the placebo arm (least-squares mean difference, 115.7 points; *P* < 0.001) favoring the patisiran arm [24, 25].

The drug effectiveness extended across the sensory-motor and autonomic domains and were consistent across patient subgroups [15].

### Safety of RNA-targeted therapies

Inotersen-related safety concerns included thrombocytopenia (< 140,000 cells/mm) in 54% of the patients in the drug arm versus 13% in the placebo arm, and three cases (3%) with glomerulonephritis. Five (4%) deaths were reported between patients in the Neuro-TTR trial, all of which occurred in the inotersen group; one death was considered possibly related to the drug [14].

The frequency of adverse events was similar between patients who received patisiran (28%) and those who received placebo (36%), most of which were mild or moderate in severity. Peripheral edema (30% vs. 22%) and infusion-related reactions (19% vs. 9%) were the adverse events that occurred more frequently with patisiran than with placebo [15]. The frequency of serious adverse events was similar between groups (36% and 40%, respectively) with four serious adverse reactions of atrioventricular heart block occurring in patients who received patisiran. Deaths were balanced between the patisiran (5%) and placebo (8%) arms of the study [15].

## Discussion

Autonomic neuropathy is an important concern in hATTR amyloidosis patients, particularly in early-onset V30M patients, being non-negligible in late-onset cases or even in other mutations associated with different phenotypes [26]. Autonomic dysfunction has been described as mainly responsible for the poor quality of life in patients and short survival of hATTR amyloidosis [12]. The presented clinical case illustrates the impact of autonomic involvement on daily living activities and quality of life of a patient with hATTRV30M early-onset amyloidosis. A significant improvement in the quality of life with stabilization of neuropathy and improvement of gastrointestinal manifestations was progressively reported by the patient throughout the treatment.

Quantification and assessment of autonomic dysfunction is a challenge to any physician, due to the low sensitivity of the available methods [16]. Compound scores are needed to address a higher significance, but a standardized approach to evaluate autonomic involvement in hATTR amyloidosis is still an unmet need in the field.

The poor response to most of the drugs used in the symptomatic management of autonomic and GI manifestations raises the need for other therapeutic approaches, in particular, disease-modifying therapies [27].

Stabilization of GI symptoms and improvement of nutritional status have been reported after liver transplantation [28], but not in autonomic manifestations [9]. Tafamidis, a TTR stabilizer, improved patient nutritional status [29, 30], but only a few data are available on the efficacy of the drug regarding the different autonomic domains [8, 29–31].

The therapeutic scenario in hATTR amyloidosis changed recently with the approval of two new TTR gene-silencing drugs, namely inotersen and patisiran. Both drugs have shown therapeutic efficacy in all domains of neuropathy, whether somatic or autonomic, as well in quality of life, being consistent in all groups and TTR mutations [14, 15].

Drug efficacy, as demonstrated by the results in the reported clinical trials, is undeniable, although the effectiveness cannot be compared directly between the two drugs due to the use of different polyneuropathy evaluation scales (mNIS+7). A good correlation between disease stage and mNIS+7 has been shown [18].

Some measures used in mNIS+7, including HRDB used in the Neuro-TTR (inotersen) study, have been recognized as having some limitations in assessing disease progression, due to a ceiling effect that prevents the recognition of worsening in subsequent evaluations. HRDB was replaced on the mNIS+7 score in the APOLLO trial by the measurement of postural blood pressure, with the rationale that HRDB has limitations for patients with pacemakers or cardiac arrhythmias. In the two scales, HRDB or postural blood pressure have a minor weighting on the total mNIS+7 score of, respectively, 3.6% and 0.7%. Regarding the efficacy of treatment in the mNIS+7 component and subcomponent scores, and in the Norfolk QOL-DN domains, the treatment effect favors both drugs in all domains. Consistent effects in favor of patisiran and inotersen were noted in Norfolk QOL-DN scores across all individual domains, namely physical functioning/larger fiber, symptoms and autonomic domains. The improvement in quallity of life at 18 months in patients on patisiran was related to strength, digestive health, ability to perform everyday activities and steadiness on their feet [23].

The nutritional status assessed by mBMI, an index of good prognosis and improved survival, evaluated in the APOLLO trial, demonstrated the efficacy of patisiran preserving nutritional status [15, 25]. The least-squares mean difference between the Neuro-TTR trial groups for BMi showed a trend in favor of inotersen to slow weight loss and preserve nutritional status [14].

COMPASS-31, an autonomic symptom assessment scale, was used as a secondary endpoint in the APOLLO trial, with results that showed a significant improvement of autonomic symptoms in the therapeutic arm, with a significant difference in relation to placebo [15].

## Conclusions

Autonomic dysfunction and gastrointestinal involvement are the main manifestations of the disease, responsible for poor quality of life and survival of hATTR amyloidosis patients. Besides the limitations presented by the different measurements used, both drugs were able to demonstrate effectiveness in halting disease progression and even offering potential improvement in the different domains of the neuropathy and quality of life.

## References

[1] Sekijima Y (2015). Transthyretin (ATTR) amyloidosis: clinical spectrum, molecular pathogenesis and disease-modifying treatments. J Neurol Neurosurg Psychiatry.

[2] Ando Y, Coelho T, Berk JL, Cruz MW, Ericzon BG, Ikeda S, Lewis WD, Obici L, Planté-Bordeneuve V, Rapezzi C, Said G, Salvi F (2013). Guideline of transthyretin-related hereditary amyloidosis for clinicians. Orphanet J Rare Dis.

[3] Gertz MA (2017). Hereditary ATTR amyloidosis: burden of illness and diagnostic challenges. Am J Manag Care.

[4] Conceição I, González-Duarte A, Obici L, Schmidt HH, Simoneau D, Ong ML, Amass L (2016). “Red-flag” symptom clusters in transthyretin familial amyloid polyneuropathy. J Peripher Nerv Syst.

[5] Suhr OB, Lundgren E, Westermark P (2017). One mutation, two distinct disease variants: unravelling the impact of transthyretin amyloid fibril composition. J Intern Med.

[6] Koike H, Nakamura T, Nishi R, Ikeda S, Kawagashira Y, Iijima M, Katsuno M, Sobue G (2018). Widespread cardiac and vasomotor autonomic dysfunction in non Val30Met hereditary transthyretin amyloidosis. Intern Med.

[7] Wang AK, Fealey RD, Gehrking TL, Low PA (2008). Patterns of neuropathy and autonomic failure in patients with amyloidosis. Mayo Clin Proc.

[8] Suhr OB, Conceição IM, Karayal ON, Mandel FS, Huertas PE, Ericzon BG (2014). Post hoc analysis of nutritional status in patients with transthyretin familial amyloid polyneuropathy: impact of tafamidis. Neurol Ther.

[9] Wixner J, Mundayat R, Karayal ON, Anan I, Karling P, Suhr OB, THAOS investigators (2014). THAOS: gastrointestinal manifestations of transthyretin amyloidosis-common complications of a rare disease. Orphanet J Rare Dis.

[10] Ikeda S, Yanagisawa N, Hongo M, Ito N (1987). Vagus nerve and celiac ganglion lesions in generalized amyloidosis. A correlative study of familial amyloid polyneuropathy and AL-amyloidosis. J Neurol Sci.

[11] Anan I, El-Salhy M, Ando Y, Nyhlin N, Terazaki H, Sakashita N, Suhr O (1999). Colonic endocrine cells in patients with familial amyloidotic polyneuropathy. J Intern Med.

[12] Gonzalez-Duarte A (2018). Autonomic involvement in hereditary transthyretin amyloidosis (hATTR amyloidosis). Clin Auton Res.

[13] Coutinho CA, Conceição I, Almeida A, Cantinho G, Sargento L, Vagueiro MC (2004). Early detection of sympathetic myocardial denervation in patients with familial amyloid polyneuropathy type I. Rev Port Cardiol.

[14] Benson MD, Waddington-Cruz M, Berk JL, Polydefkis M, Dyck PJ, Wang AK, Planté-Bordeneuve V, Barroso FA, Merlini G, Obici L, Scheinberg M, Brannagan TH, Litchy WJ, Whelan C, Drachman BM, Adams D, Heitner SB, Conceição I, Schmidt HH, Vita G, Campistol JM, Gamez J, Gorevic PD, Gane E, Shah AM, Solomon SD, Monia BP, Hughes SG, Kwoh TJ, McEvoy BW, Jung SW, Baker BF, Ackermann EJ, Gertz MA, Coelho T (2018). Inotersen treatment for patients with hereditary transthyretin amyloidosis. New Engl J Med.

[15] Adams D, Gonzalez-Duarte A, O’Riordan WD, Yang CC, Ueda M, Kristen AV, Tournev I, Schmidt HH, Coelho T, Berk JL, Lin KP, Vita G, Attarian S, Planté-Bordeneuve V, Mezei MM, Campistol JM, Buades J, Brannagan TH, Kim BJ, Oh J, Parman Y, Sekijima Y, Hawkins PN, Solomon SD, Polydefkis M, Dyck PJ, Gandhi PJ, Goyal S, Chen J, Strahs AL, Nochur SV, Sweetser MT, Garg PP, Vaishnaw AK, Gollob JA, Suhr OB (2018). Patisiran, an RNAi therapeutic, for hereditary transthyretin amyloidosis. N Engl J Med.

[16] Novak P (2011). Quantitative autonomic testing. J Vis Exp.

[17] Dyck PJ, Kincaid JC, Dyck PJB, Chaudhry V, Goyal NA, Alves C, Salhi H, Wiesman JF, Labeyrie C, Robinson-Papp J, Cardoso M, Laura M, Ruzhansky K, Cortese A, Brannagan TH, Khoury J, Khella S, Waddington-Cruz M, Ferreira J, Wang AK, Pinto MV, Ayache SS, Benson MD, Berk JL, Coelho T, Polydefkis M, Gorevic P, Adams DH, Plante-Bordeneuve V, Whelan C, Merlini G, Heitner S, Drachman BM, Conceição I, Klein CJ, Gertz MA, Ackermann EJ, Hughes SG, Mauermann ML, Bergemann R, Lodermeier KA, Davies JL, Carter RE, Litchy WJ (2017). Assessing mNIS+7 ionis and international neurologists’ proficiency in a familial amyloidotic polyneuropathy trial. Muscle Nerve.

[18] Adams D, Suhr OB, Dyck PJ, Litchy WJ, Leahy RG, Chen J, Gollob J, Coelho T (2017). Trial design and rationale for APOLLO, a phase 3, placebo controlled study of patisiran in patients with hereditary ATTR amyloidosis with polyneuropathy. BMC Neurol.

[19] Coelho T, Vinik A, Vinik EJ, Tripp T, Packman J, Grogan DR (2017). Clinical measures in transthyretin familial amyloid polyneuropathy. Muscle Nerve.

[20] Vinik EJ, Vinik AI, Paulson JF, Merkies IS, Packman J, Grogan DR, Coelho T (2014). Norfolk QOL-DN: validation of a patient reported outcome measure in transthyretin familial amyloid polyneuropathy. J Peripher Nerv Syst.

[21] Treister R, O’Neil K, Downs HM, Oaklander AL (2015). Validation of the composite autonomic symptom scale 31 (COMPASS-31) in patients with and without small fiber polyneuropathy. Eur J Neurol.

[22] Suhr O, Danielsson A, Holmgren G, Steen L (1994). Malnutrition and gastrointestinal dysfunction as prognostic factors for survival in familial amyloidotic polyneuropathy. J Intern Med.

[23] Obici L, Coelho T, Gonzalez-Duarte A (2018). Impact of Patisiran on Norfolk Quality of Life Questionnaire diabetic neuropathy (QOL-DN) in patients with hereditary transthyretin-mediated amyloidosis: results from the phase 3 APOLLO study. Eur J Neurol.

[24] Kristen AV, Ajroud-Driss S, Conceição I, Gorevic P, Kyriakides T, Obici L (2018). Patisiran, an RNAi therapeutic for the treatment of hereditary transthyretin-mediated amyloidosis. Neurodegener Dis Manag.

[25] Obici L, Coelho T, Adams D, Gonzalez-Duarte A, O’Riordan W, Yang C-C, Yamashita T, Kristen A, Tournev I, Schmidt H, Berk J, Lin K-P, Gandhi P, Sweetser M, Chen J, Goyal S, Gollob J, Suhr O (2018). Impact of patisiran, an investigational RNAi therapeutic, on nutritional status in patients with hereditary transthyretin-mediated amyloidosis. Eur J Neurol.

[26] Koike H, Nakamura T, Hashizume A, Nishi R, Ikeda S, Kawagashira Y, Iijima M, Katsuno M, Sobue G (2017). Cardiac and peripheral vasomotor autonomic functions in late-onset transthyretin Val30Met familial amyloid polyneuropathy. J Neurol.

[27] Wixner J, Suhr OB, Anan I (2018). Management of gastrointestinal complications in hereditary transthyretin amyloidosis: a single-center experience over 40 years. Expert Rev Gastroenterol Hepatol.

[28] Lång K, Wikström L, Danielsson A, Tashima K, Suhr OB (2000). Outcome of gastrointestinal complications after liver transplantation for familial amyloidotic polyneuropathy. Scand J Gastroenterol.

[29] Coelho T, Maia LF, Martins da Silva A, Waddington Cruz M, Planté-Bordeneuve V, Lozeron P, Suhr O, Campistol JM, Conceição I, Schmidt HJ, Trigo P, Kelly J, Labaudinière R, Chan J, Packman J, Wilson A, Grogan DR (2012). Tafamidis for transthyretin familial amyloid polyneuropathy: a randomized, controlled trial. Neurology.

[30] Coelho T, Maia LF, da Silva AM, Cruz MW, Planté-Bordeneuve V, Suhr OB, Conceiçao I, Schmidt HH, Trigo P, Kelly JW, Labaudinière R, Chan J, Packman J, Grogan DR (2013). Long-term effects of tafamidis for the treatment of transthyretin familial amyloid polyneuropathy. J Neurol.

[31] Cortese A, Vita G, Luigetti M (2016). Monitoring effectiveness and safety of tafamidis in a transthyretin amyloidosis in Italy: a longitudinal multicenter study in a non-endemic area. J Neurol.

